# Antiobesity Effect of *Lacticaseibacillus paracasei* LM-141 on High-Fat Diet-Induced Rats through Alleviation of Inflammation and Insulin Resistance

**DOI:** 10.1155/2023/1011591

**Published:** 2023-04-18

**Authors:** Ching-Shuang Wu, Chih-Chieh Lin, Feng-Ching Hsieh, Tai-Yun Wu, Ai-Hui Fang

**Affiliations:** ^1^Department of Medical Laboratory Science and Biotechnology, Kaohsiung Medical University, Kaohsiung 80708, Taiwan; ^2^Institute of Biomedical Sciences, National Sun Yat-Sen University, Kaohsiung 80708, Taiwan; ^3^Larmood Company Limited, Pingtung 90076, Taiwan; ^4^Department of General Medicine, Tri-Service General Hospital, National Defense Medical Center, Taipei 11466, Taiwan; ^5^Department of Microbiology, College of Medicine, Kaohsiung Medical University, Kaohsiung 80708, Taiwan

## Abstract

In this study, we set out to evaluate the antiobesity activities of our newly isolated *Lacticaseibacillus paracasei* LM-141 (LPLM141) using a high-fat diet (HFD)-fed rat model. Male Sprague–Dawley rats were fed with a HFD with or without low-dosage (2 × 10^7^ CFU/day per rat) or high-dosage (2 × 10^9^ CFU/day per rat) LPLM141 for 14 weeks. The results showed that administration of LPLM141 significantly decreased body weight gain, liver weight, adipose tissue weight, and epididymal white adipocyte size increased by HFD feeding. The abnormal serum lipid profile induced by HFD feeding was normalized by administration of LPLM141. The enhanced chronic low-grade inflammation in HFD-fed rats was reduced by LPLM141 supplementation, as reflected by decreased serum lipopolysaccharide (LPS) and monocyte chemoattractant protein-1 (MCP-1) levels, reduced macrophage infiltration in adipose tissue, and increased serum adiponectin concentration. In addition, the elevations of proinflammatory cytokine genes and suppression of PPAR-*γ* mRNA in adipose tissues of rats fed with a HFD were markedly reversed by LPLM141 administration. Oral administration of LPLM141 induced browning of epididymal white adipose tissue (eWAT) and activation of interscapular brown adipose tissue (iBAT) in rats fed with HFD. Consumption of LPLM141 exhibited a significant amelioration in insulin resistance, which were mechanistically caused by downregulation of the serum leptin level and upregulation of hepatic IRS-1 and p-Akt protein expressions, in HFD treated rats. LPLM141 consumption significantly decreased hepatic lipogenic gene expressions and preserved liver function stimulated by HFD treatment. Administration of LPLM141 obviously mitigated hepatic steatosis observed in HFD feeding rats. Our current findings shed light on LPLM141 supplementation that exhibited an antiobesity effect in HFD-fed rats by alleviating inflammation and insulin resistance, which further highlighted the potential of utilizing LPLM141 as a preventive/therapeutic probiotic agent for obesity.

## 1. Introduction

Metabolic syndrome is a cluster of metabolic disorders including hyperglycemia, hyperinsulinemia, and hyperlipidemia, resulting from metabolic dysregulation [1]. These metabolic disorders are considered to be critical contributors to obesity-related diseases, such as type 2 diabetes, insulin resistance, and fatty liver disease [2]. Obesity is a complicated disease characterized by accumulation of lipids in metabolic tissues, mainly adipose tissue and the liver, and is a serious public health issue in the whole world in view of its morbidity and mortality [3]. According to the report from the World Health Organization [4], worldwide obesity has nearly tripled since 1975 and approximately 39% of adults are now overweight and 13% are obese in 2016. Therefore, it is imperative to study the available strategy that can assist with preventing and treating obesity.

Over several decades, the habitual consumption toward high fat and/or high glucose of western-type diets significantly increases the prevalence of obesity [5]. More specifically, accumulating evidences demonstrate that the excess accumulation of body fat frequently causes the increase of chronic low-grade inflammation in obesity [6]. In addition, previous studies also suggested that the chronic low-grade inflammation in obesity can increase macrophage infiltration and proinflammatory adipokine secretion, including interleukin (IL)-1, -6 and tumor necrosis factor (TNF)-*α* by adipose tissues, which interfere with the insulin-signaling pathway in related tissues and ultimately lead to the development of insulin resistance and the subsequent progression to type 2 diabetes [7, 8]. Thus, the persistent chronic low-grade inflammation might be one of the key points for the development of obesity and insulin resistance, which can be regarded as the target for the screening of antiobesity supplementations.

More and more studies indicate that the intestinal microbiota play a crucial role in the development of obesity [9, 10]. Gut microbiota have been reported to regulate energy metabolic balance and inflammatory status by acting on intestinal integrity [11, 12]. The impairment of gut integrity causes upregulation of the systemic endotoxin level and further enhances chronic low-grade inflammation, thereby promoting the development of obesity. These results raise the possibility that the intentional manipulation of the community structure of gut microbiota may be a potential strategy to treat obesity. As an essential component of gut microbiota, probiotics gradually emerge as beneficial microbes to human health through various mechanisms including modulation of the community structure of gut microbiota, modification of microenvironment with the gut, increase of the gut epithelial barrier function, balancing of energy homeostasis, and regulation of the host immune response [13]. A large body of evidences has demonstrated that the best way for controlling the flora balance in intestine is achieved by intake of probiotics, which is thought to be effective in treating obesity [14, 15]. Probiotics are defined as viable nonpathogenic microorganisms that confer health benefits to the host when ingested in adequate amount as food ingredients. Among probiotics, *Lacticaseibacillus* spp. and *Bifidobacterium* spp. are the most relevant in ameliorating obesity and improving metabolic parameters [16]. Nevertheless, not all discovered probiotics possess health-promoting or antiobesogenic effects, some results indicate no health benefits or even obesogenic effects associated with probiotics [17–19]. This leads to the existence of species-specific effects with different mechanistic actions of probiotics on obesity or other metabolic disorders' improvement. Therefore, it is necessary and imperative to carefully screen out the probiotic strains with higher efficacy in obesity management.

Recently, we isolated several strains of Lactobacillus spp., including *Lacticaseibacillus rhamnosus*, *Lacticaseibacillus plantarum*, *Lacticaseibacillus paracasei*, *Lacticaseibacillus casei*, and *Lactobacillus delbrueckii* (*subsp. bulgaricus*) from Taiwanese kimchi, breast milk, newborn feces, and Tempeh (a kind of Indonesian fermented soy food). These isolated Lactobacillus strains were then identified by 16S rRNA gene sequencing, followed by the ribotyping technique [20]. As aforementioned, obesity is characterized by a chronic low-grade inflammation that may affect the insulin activity in its metabolically sensitive tissues. Therefore, the anti-inflammatory ability is considered to be the most important criterion for strain selection. After coculture of murine macrophages with our isolated *Lactobacillus strains*, *Lacticaseibacillus paracasei* LM-141 (LPLM141) exhibited the best anti-inflammatory effect, as shown by the highest interleukin 10 (IL-10) versus IL-6 (IL-10/IL-6; anti-/proinflammatory) secretion ratio. The present study aimed to explore whether the selected probiotic in terms of LPLM141 may reduce obesity, inflammation, and insulin resistance in rats fed with a high-fat diet (HFD).

## 2. Materials and Methods

### 2.1. Preparation of *Lacticaseibacillus paracasei* LM-141 (LPLM141)

LPLM141 cells were prepared according to previous protocols [21]. After centrifugation, LPLM141 cells were washed with sterile 0.85% NaCl solution followed by lyophilizing and then kept at −80°C until use. The amount of LPLM141 cells in lyophilized powder was higher than 10^11^ cells/gram. The lyophilized powder containing LPLM141 cells was dissolved with sterile RO water and orally administered to the rats.

### 2.2. Animal Model

The animal study was approved by the Institutional Animal Care and Use Committee (IACUC) of Kaohsiung Medical University (approval no. 108019) and conducted according to the guidelines laid down by the IACUC. Five-week-old pathogen-free male Sprague–Dawley (SD) rats were purchased from BioLASCO (BioLASCO Taiwan Co., Ltd, Yi-Lan, Taiwan) and housed at 24 ± 1°C, 50% humidity, and 12 h light-dark cycles with free access to water and food.

### 2.3. Experimental Design

After one week of acclimatization, a total of thirty-two male rats were randomly divided into four groups (*n* = 8/group) as follows: (1) the control group (control) consumed a standard diet; (2) the high-fat diet (HFD) group consumed a high-fat diet (a diet containing 59.3% energy from fat); (3) the HFD + low-dosage group that consumed a high-fat diet was fed with low-dosage LPLM141 (2 × 10^7^ CFU/day per rat); and (4) the HFD + high-dosage group that consumed a high-fat diet was fed with high-dosage LPLM141 (2 × 10^9^ CFU/day per rat). Five weeks after feeding with standard diet or high-fat diet, the rats were administered with extra RO water (for control and HFD groups) or different dosages of LPLM141 (for HFD + low- or high-dosage groups) till the end of experiments. The oral gavage was performed at fixed time every day. The composition of the standard diet and the high-fat diet is listed in Table 1. Body weight was recorded twice a week, and diet intake was recorded once a week. At the end of the experiments (14 weeks), the rats were fasted overnight before sacrificing.

### 2.4. Blood and Tissue Collection

Blood and tissue samples were collected from animals sacrificed by CO_2_ anesthesia at the end of the experiments. The blood samples were collected in a sterile tube by cardiac puncture. After having been centrifuged at 4,000*g* for 10 min at 4°C, the serum was collected and stored at −80°C for further experiments. Tissue samples including the liver, kidney, and different adipose tissues (including subcutaneous white adipose tissue (sWAT), epididymal white adipose tissue (eWAT), and interscapular brown adipose tissue (iBAT)) were weighed and washed with saline and then stored at −80°C until use.

### 2.5. Oral Glucose Tolerance Test (OGTT)

Thirteen weeks after treatment, the oral glucose tolerance test was performed. After fasting for 12 h, an oral glucose load (2 g/body weight (kg)) was given to each rat. Blood samples collected from the tail vein at 0, 30, 60, 90, and 120 min after glucose administration were subjected to glucose level determinations using a glucometer Rightest GM550 (Bionime, Taichung, Taiwan).

### 2.6. Serum Biochemical Parameter Evaluation

Serum biochemical parameters including TG, LDL-C, HDL-C, cholesterol, AST, and ALT were analyzed using an automatic clinical analyzer (Hitachi High-Technologies Corporation, Tokyo, Japan). Serum glucose concentrations were determined using a glucometer Rightest GM550 (Bionime). Serum levels of insulin, adiponectin, leptin, LPS, and MCP-1 were measured using commercial rat ELISA kits (Invitrogen, Carlsbad, CA, USA) according to the manufacturer's instructions.

### 2.7. Analysis of Gene Expression by Real-Time RT-PCR

Total RNA of eWAT (for TNF-*α*, IL-6, IL-1*β*, peroxisome proliferator-activated receptor-*γ* (PPAR-*γ*), uncoupling protein-1 (Ucp-1), PR domain-containing protein-16 (Prdm16), peroxisome proliferator-activated receptor-*γ*-coactivator-1*α* (PGC-1*α*), transmembrane protein 26 (Tmen26), CD137), sWAT (for TNF-*α*, IL-6, IL-1*β*, PPAR-*γ*), iBAT (for Ucp-1, Prdm16, PGC-1*α*, cell death-inducing DFFA-like effector a (Cidea)), and hepatic tissue (for elongation of very long-chain fatty acids protein 6 (Elvol6), fatty acid synthase (FAS), and sterol regulatory element-binding transcription factor 1c (Srebp-1c)) of each rat were extracted using TRI reagent (Invitrogen, Carlsbad, CA, USA). Then, the real-time RT-PCR was performed using SYBR Green Master Mix (Roche Applied Science, Indianapolis, IN, USA) on ABI Prism 7500 Sequence Detection System (Applied Biosystems). The sequences of the primers used for real-time RT-PCR are listed in Table 2. The relative gene expression was determined by the 2^−ΔΔCt^ method using *β*-actin as the reference gene.

### 2.8. Western Blotting Analysis

Western blotting was performed to analyze the expressions of insulin receptor substrate-1 (IRS-1), total Akt, phosphor-AKT, and *β*-actin. Five to seven mg of rat liver tissue was homogenized in an ice-cold RIPA buffer (1% Triton, 0.1% SDS, 0.5% deoxycholate, 1 mmol/L EDTA, 20 mmol/L Tris (pH 7.4), 150 mmol/L NaCl, 10 mmol/L NaF, and 0.1 mmol/L phenylmethylsulfonyl fluoride). The homogenate was centrifuged at 14,000 × *g* for 20 min at 4°C to remove debris. The resulting supernatant was retained as the total cellular protein lysate. Fifty *μ*g of total cellular proteins were loaded and subjected to electrophoresis for protein separation. The resolved proteins were transferred to a PVDF membrane (Biomate, Kaohsiung, Taiwan) and then were incubated with the blocking buffer for 2 h. The membranes were incubated with primary antibodies (*β*-actin (1 : 1,000 dilution, v/v), IRS-1 (1 : 500 dilution, v/v)) (Elabscience, Houston, USA), phospho-Akt Ser473 (1 : 500 dilution, v/v), and total Akt (1 : 500 dilution, v/v) (Cell Signaling Technology, Massachusetts, USA) overnight at 4°C. Subsequently, membranes were incubated with the corresponding secondary antibody (Goat anti-rabbit IgG HRP, 1 : 10,000 dilution, v/v) (Santa Cruz Biotechnology, Dallas, USA) for 2 h at room temperature. Membranes were washed three times with 0.1% PBST for 30 min. The protein bands were visualized by T-Pro Lumilong Plus Chemiluminescent Substrate kit (T-Pro Biotechnology, New Taipei County, Taiwan) and scanned using Chemi I luminescent Imaging System (Mini Chemi I; SageCreation Science, Beijing, China).

### 2.9. Histological Studies

Both the rat hepatic and eWAT were fixed in 10% neutral -buffered formalin at 4°C for two days and then were frozen in Tissue-Tek. The liver sections (8 *μ*m thick) were rehydrated and stained with oil red O (Sigma-Aldrich, St. Louis, MO, USA) with Mayer's hematoxylin as counterstaining [22]. For hematoxylin and eosin (H&E) staining, the liver and eWAT isolated from rats were fixed in 10% formalin and embedded in paraffin. Three micrometer thick sections were deparaffinized in xylene and stained with hematoxylin-eosin. The images of staining were viewed and captured with an Olympus microscope (Olympus Corporation, Tokyo, Japan). Image J software (National Institutes of Health, USA) was used to calculate adipocyte size.

### 2.10. Immunohistochemical Staining

eWATs derived from the rats were fixed in 10% neutral-buffered formalin for 72 h and were dehydrated and paraffin embedded. The sections (4 *μ*m thick) were stained with anti-F4/80 antibodies (dilution 1 : 100) (Nichirei, Tokyo, Japan) overnight at 4°C. Then, the sections were reacted with a secondary antibody of Histofine Simple Stain Max PO (rat) (Nichirei) for 30 min. The images were captured using a Zeiss Stallion Dual Detector Imaging System (Carl Zeiss Microimaging Inc., NY, USA).

### 2.11. Statistical Analysis

The data were presented as the mean ± SEM. Statistical analyses were performed by *Student*'*s t-test* when two groups were compared and by one-way ANOVA followed by Tukey's test procedures using SPSS 20.0 (SPSS Inc., Chicago, IL, USA) when more than two groups were compared. All the assays were obtained from at least three independent experiments. A *p* value < 0.05 was considered statistically significant.

## 3. Results

### 3.1. Administration of *Lacticaseibacillus paracasei* LM-141 (LPLM141) Mitigates Obesity and Lipid Accumulation in High-Fat Diet-Fed Rats

**Figure 1:**
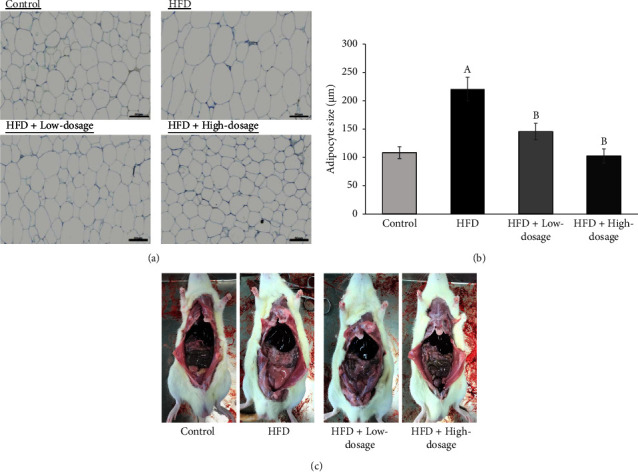
Effects of LPLM141 administration on (a) hematoxylin and eosin staining of epididymal adipose tissue; (b) quantification of epididymal adipocyte size; and (c) the anatomical image of abdominal fat accumulation in rats. Scale bar = 60 *μ*m. Data are expressed as the mean ± SEM. ^(A-B)^Values of bar with different letters differ significantly at *p* < 0.05. ^(A)^HFD versus control; ^(B)^HFD + low dosage and HFD + high dosage versus HFD.

At the beginning of the experiment, the initial body weight among different groups showed no significant difference. The body weight and body weight gain in the HFD group was significantly increased as compared to the control group at the 14th week, while this increase was downregulated by the administration of low- and high-dosage LPLM141. Of note, the food intake showed no significant difference among all groups, indicating that the effects of LPLM141 on body weight were not due to the changes in appetite (Table 3). In addition, compared to the control group, the liver and the subcutaneous and eWAT weights, which were significantly increased in the HFD group, were markedly decreased after oral low- or high-dosage LPLM141 treatment. However, the iBAT weight was not significantly different among different groups. There were no significant differences in kidney and spleen weights among different groups (Table 3). In addition to decrease body weights and some tissue weights' (liver and adipose tissues) increase by the high-fat diet after the administration of LPLM141, the average epididymal adipocyte size was also significantly reduced by LPLM141 supplementation as compared to the HFD group (Figures 1(a) and 1(b)). The anatomical images of abdominal fat accumulation shown in Figure 1(c) further corroborated that LPLM141 intervention effectively improved obesity and lipid accumulation in HFD-fed rats.

### 3.2. *Lacticaseibacillus paracasei* LM-141 Improves Serum Lipid Profiles in High-Fat Diet-Fed Rats

**Figure 2:**
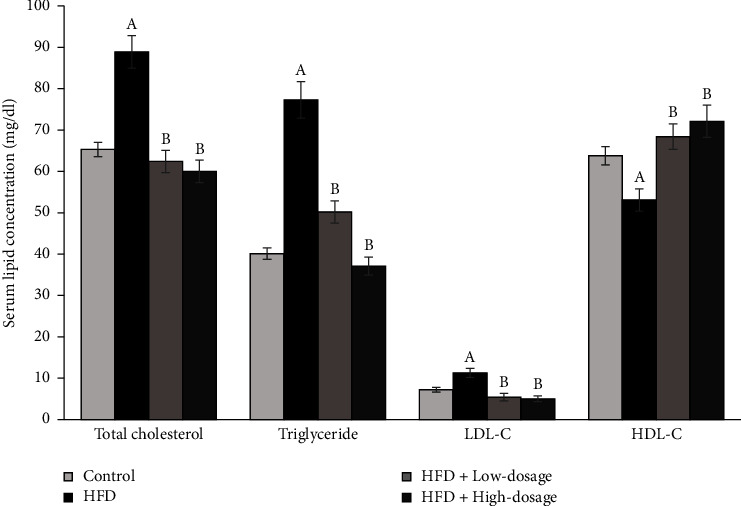
Effects of LPLM141 administration on serum lipid profiles in rats fed with a high-fat diet for 14 weeks. Data are expressed as the mean ± SEM. ^(A-B)^Values of bar with different letters differ significantly at *p* < 0.05. ^(A)^HFD versus control; ^(B)^HFD + low dosage and HFD + high dosage versus HFD.

It is well known that the excessive fat accumulation often accompanies metabolic disorders, so the levels of serum TC, TG, LDL-C, and HDL-C were detected. The results demonstrated in Figure 2 revealed that an increase in the serum TC, TG, and LDL-C levels and a decrease in HDL-C levels were detected in the HFD group when compared with the control group. However, both low- and high-dosage LPLM141 supplementation significantly reduced serum TC, TG, and LDL-C levels and increased serum HDL-C levels as compared to the HFD group.

### 3.3. *Lacticaseibacillus paracasei* LM-141 Suppresses Inflammation in High-Fat Diet-Fed Rats

**Figure 3:**
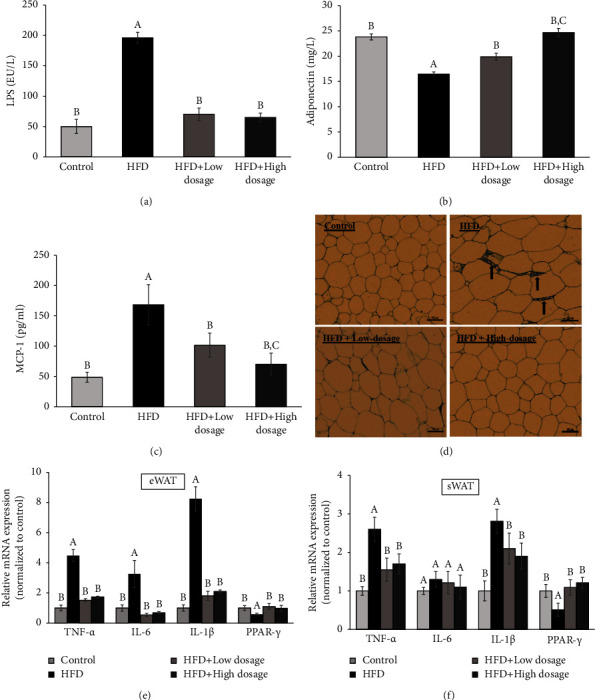
Effects of LPLM141 administration on the state of inflammation in rats fed with a high-fat diet. (a) Circulating level of LPS; (b) serum adiponectin concentration; (c) serum MCP-1 level; and (d) F4/80 immunostaining of epididymal adipose tissue. Black arrows indicate crown-like structure of adipocytes with infiltrated macrophages. Scale bar = 60 *μ*m; Inflammatory-associated gene expressions in epididymal (e) and subcutaneous (f) white adipose tissues. Data are expressed as the mean ± SEM. ^(A–C)^Values of bar with different letters differ significantly at *p* < 0.05. ^(A)^HFD versus control; ^(B)^HFD + low dosage and HFD + high dosage versus HFD; and ^(C)^HFD + high dosage versus HFD + low dosage.

Chronic low-grade inflammation is an important character associated with obesity. It was evidenced that LPS derived from Gram-negative bacteria in the gut played a critical role in the development of tissue inflammation in obesity [23]. The result shown in Figure 3(a) demonstrated that a significant higher serum LPS level was observed in the HFD group compared with the control group. LPLM141 administration could effectively decrease LPS concentration which was increased in the HFD group. Furthermore, the serum level of anti-inflammatory adipokine, namely, adiponectin was decreased in the HFD group as compared to the control group. However, both low- and high-dosage LPLM141 interventions significantly upregulated serum adiponectin concentrations reduced by high-fat diet treatment (Figure 3(b)). MCP-1 is a proinflammatory chemokine that recruits and activates macrophages in adipose tissues of obese animals [24]. Our present results indicated that the serum level of MCP-1 in the HFD group was significantly elevated compared with the control group. However, both low- and high-dosage LPLM141 supplementation reduced the serum MCP-1 level in rats fed with a high-fat diet (Figure 3(c)). We further examined the infiltration of macrophages in adipose tissue by immunohistochemical analysis with an anti-F4/80 antibody to evaluate the involvement of adipose tissue inflammation. The results shown in Figure 3(d) demonstrated that a significant percentage of macrophage infiltration in adipocytes was observed in the HFD group (42.3 ± 3.2%) when compared with the control group (19.7 ± 1.4%). However, the HFD-induced infiltration of macrophages in the adipocytes was significantly inhibited by both low- and high-dosage of LPLM141 treatment (30.0 ± 2.5% and 11.1 ± 1.2%, respectively) (Figure 3(d)). It is generally recognized that the inflammatory response emerging in the presence of obesity is predominantly attributed to the inflammation in adipose tissue, although other metabolically critical sites may also be involved [25]. Therefore, we next monitored mRNA expression levels of proinflammatory cytokines in epididymal and subcutaneous adipose tissues after high-fat diet feeding with or without LPLM141 administration. As shown in Figure 3(e), TNF-*α*, IL-6, and IL-1*β* mRNA expression levels in eWAT were higher in the HFD group compared with the control group. Both low- and high-dosage LPLM141 supplementation significantly reduced cytokine expression levels compared with the HFD group. Similar to the results demonstrated in eWAT (Figure 3(e)), the upregulation of TNF-*α* and IL-1*β* mRNA expressions in sWAT after HFD feeding were downregulated by both low- and high-dosage LPLM141 treatments (Figure 3(f)). Although not statistically significant, the increased IL-6 mRNA expression induced by HFD in subcutaneous adipose tissue was decreased by both low- and high-dosage LPLM141 administration (Figure 3(f)). PPAR-*γ*, highly expressed in adipose tissue, is known to decrease adipose tissue inflammation by decreasing inflammatory gene expression [26, 27]. As demonstrated in Figures 3(e) and 3(f), high-fat diet treatment significantly suppressed PPAR-*γ* mRNA expression in epididymal and subcutaneous adipose tissue compared with the control group. Both low- and high-dosage LPLM141 administration increased PPAR-*γ* mRNA expression which was decreased in the HFD group. Based on these results, we concluded that LPLM141 administration effectively alleviated inflammation by decreasing the serum LPS level, TNF-*α*, IL-6, and IL-1*β* mRNA expression and infiltrated macrophages in adipose tissues, increasing serum adiponectin concentration and PPAR-*γ* mRNA in adipose tissues.

### 3.4. *Lacticaseibacillus paracasei* LM-141 Administration Induces Browning in eWAT and Activation in iBAT in High-Fat Diet-Fed Rats

**Figure 4:**
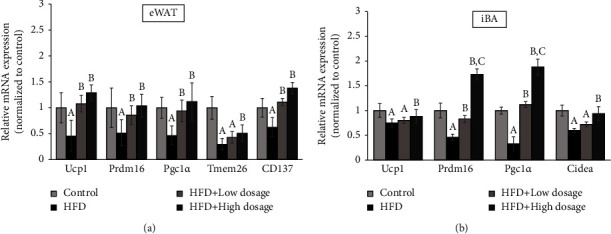
Effects of LPLM141 intervention on WAT browning and iBAT activation in high-fat diet-fed rats. (a) Relative mRNA expression of browning markers in eWAT; (b) relative mRNA expression of activation markers in iBAT. Data are expressed as the mean ± SEM. ^(A–C)^Values of bar with different letters differ significantly at *p* < 0.05. ^(A)^HFD and/or HFD + low dosage versus control; ^(B)^HFD + high dosage and/or HFD + low dosage versus HFD; and ^(C)^HFD + high dosage versus HFD + low dosage.

In addition to its role in regulating tissue inflammation, PPAR-*γ* was also considered the master regulator as well as a critical determinant of adipocyte differentiation and activation [28]. Since both low- and high-dosage LPLM141 treatments markedly increased PPAR-*γ* gene expression decreased by HFD feeding in white adipose tissues (Figure 3), we next set out to assess mRNA expression levels of PPAR-*γ* downstream genes related to browning in eWAT and activation in iBAT. The results shown in Figure 4(a) indicated that the levels of brown adipocyte marker genes including UCP1, Prdm16, and Pgc1*α* in eWAT were significantly reduced in the HFD group as compared to the control group. These reducing mRNA expression levels of brown adipocyte markers were recovered after LPLM141 administration. Besides, the mRNA expressions of two beige adipocyte markers, namely, Tmem26 and CD137 decreased by HFD feeding, were increased by LPLM141 treatment. We also evaluated whether the increased PPAR-*γ* mRNA expression by LPLM141 intervention in WAT could influence the activation of iBAT. The results demonstrated that HFD significantly suppressed the mRNA expression levels of known BAT markers including Ucp1, Prdm16, Pgc1*α*, and Cidea in iBAT. However, the administration of LPLM141 mitigated the decrease of the mRNA expression levels (Figure 4(b)). These results suggested that the upregulation of PPAR-*γ* after LPLM141 intervention may promote WAT browning and increase BAT activity.

### 3.5. *Lacticaseibacillus paracasei* LM-141 Administration Attenuates Insulin Resistance and Improves Glucose Tolerance in Rats Fed with High-Fat Diet

**Figure 5:**
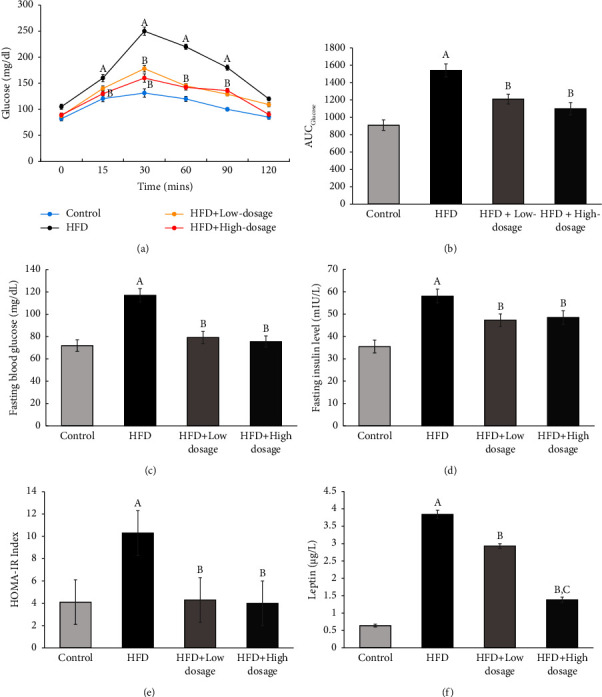
Effects of LPLM141 administration on glucose tolerance and insulin resistance in high-fat diet-fed rats. (a) Oral glucose tolerance test (OGTT) was performed at 0, 15, 30, 60, 90, and 120 min after glucose administration. The blood glucose concentrations in rat tail veins were determined by using a glucometer; (b) area under curve (AUC) analyses for glucose tolerance test at week 14; (c) fasting blood glucose level; (d) fasting insulin level; (e) HOMA-IR index at week 14; and (f) serum leptin concentration. Data are expressed as the mean ± SEM. ^(A–C)^Values of bar with different letters differ significantly at *p* < 0.05. ^(A)^HFD versus control; ^(B)^HFD + low dosage and HFD + high dosage versus HFD; and ^(C)^HFD + high dosage versus HFD + low dosage.

It is evidenced that inflammation is a triggering factor in the development of insulin resistance [27]. Therefore, it is reasonable to explore the effects of LPLM141 administration on insulin resistance induced by high-fat diet feeding since our aforementioned results validated the anti-inflammatory effects of LPLM141 intervention. We next conducted OGTT and HOMA-IR to verify the insulin sensitivity by LPLM141. As shown in Figures 5(a) and 5(b), it was apparent that impaired glucose tolerance in the HFD group compared with the control group was noticed, which was clearly verified by a higher AUC, while both low- and high-dosage LPLM141 supplementation significantly improved this impairment (Figure 5(b)). HOMA-IR is calculated by fasting glucose and insulin levels, and the results shown in Figures 5(c) and 5(d) revealed that both fasting blood glucose and fasting insulin levels were significantly higher in the HFD group compared to the control group. This enhancing effect was abrogated by the intervention of both low- and high-dosage LPLM141. Furthermore, the results showed that the value of the HOMA-IR index in the HFD group was approximately 2.5-fold higher than the control group (Figure 5(e)), while the value of HOMA-IR in the HFD + low-dosage LPLM141 group and the HFD + high-dosage LPLM141 group both exhibited an apparent reduction compared to the HFD group (Figure 5(e)). As a member of adipokines, leptin can act as a proinflammatory cytokine and play some role in the development of obesity and insulin resistance [29, 30]. Our present findings indicated that the serum level of leptin in the HFD group was significantly upregulated when compared to the control group. However, the elevated serum leptin concentration induced by high-fat diet feeding was markedly reduced by administration of both low- and high-dosage LPLM141 (Figure 5(f)).

**Figure 6:**
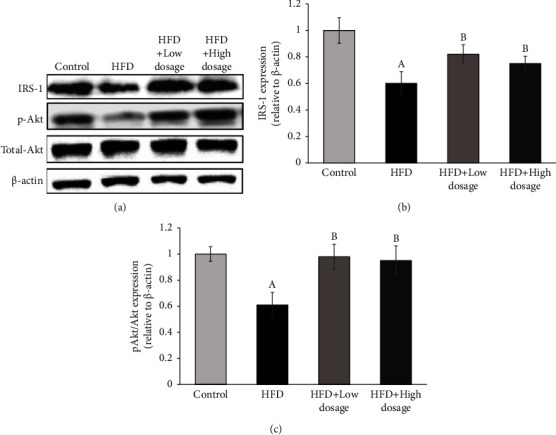
Effects of LPLM141 administration on insulin signaling-associated molecules expressions in rats fed with a high-fat diet. (a) Expressions of IRS-1, p-Akt, and total Akt in rat hepatic tissues by Western blot analysis; (b) densitometric analysis of IRS-1 expression with data normalized to the control group; and (c) densitometric quantification of p-Akt vs. total Akt expression with data normalized to the control group. Data are expressed as the mean ± SEM. ^(A-B)^Values of bar with different letters differ significantly at *p* < 0.05. ^(A)^HFD versus control; ^(B)^HFD + low dosage and HFD + high dosage versus HFD.

In order to clarify the possible mechanisms underlying the development of insulin resistance induced by high-fat diet treatment, we determined the relevant molecule expression on the insulin signaling pathway using Western blotting analysis. The results shown in Figure 6(a) indicated that both IRS-1 and phosphorylated Akt at Ser473 (p-Akt^Ser473^) protein expressions in hepatic tissues were reduced in the HFD group compared with the control group. Notably, both low- and high-dosage LPLM141 supplementation obviously reversed this inhibitory effect affected by high-fat diet feeding. The densitometry analysis of IRS-1 and p-Akt^Ser473^ derived from the blotting plot is shown in Figures 6(b) and 6(c), respectively. These results clearly demonstrated that LPLM141 intervention mitigated insulin resistance in obese rats.

### 3.6. *Lacticaseibacillus paracasei* LM-141 Ameliorates Hepatic Lipid Accumulation and Liver Damage in Rats Fed with High-Fat Diet

**Figure 7:**
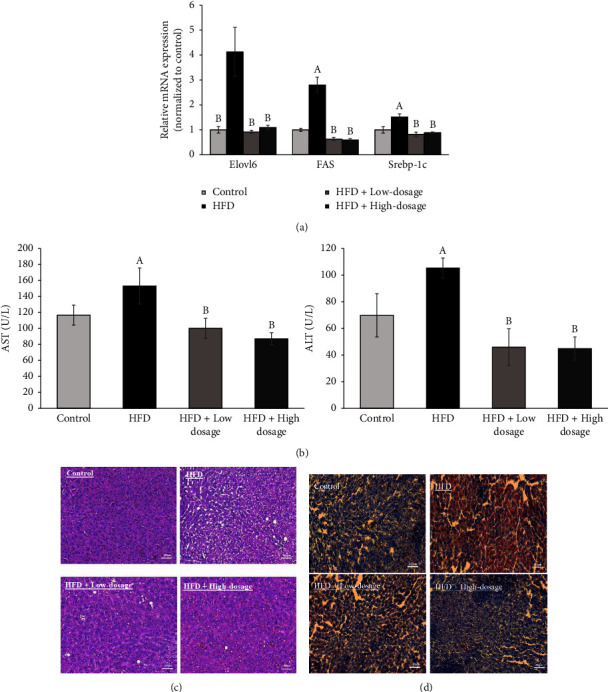
Effects of LPLM141 administration on hepatic steatosis and hepatic function in rats fed with a high-fat diet. (a) Representative mRNA expression of Elovl6, FAS, and Srebp-1c in hepatic tissues of rats; (b) serum liver function markers (AST and ALT) concentrations; (c) histological analysis of representative hematoxylin and eosin staining on the liver sections of rats; and (d) representative oil red O staining for fat deposition measurement in the liver. Scale bar = 100 *μ*m. Data are expressed as the mean ± SEM. ^(A-B)^Values of bar with different letters differ significantly at *p* < 0.05. ^(A)^HFD versus control; ^(B)^HFD + low dosage and HFD + high dosage versus HFD.

Obesity is a well-known risk factor for the development of nonalcoholic fatty liver disease (NAFLD) characterized by steatosis, inflammation, hepatocellular ballooning, and fibrosis [31]. The findings revealed in Figure 7(a) demonstrated that the mRNA expressions of hepatic lipogenic genes, namely, Elovl6, FAS, and SREBP-1c, in hepatic tissues were significantly higher in the HFD group than those in the control group, while the expression of these genes were reduced via administration of both low- and high-dosage LPLM141. In addition, two important liver function markers, namely, ALT and AST, were significantly elevated in the serum of the HFD group rats as compared to those of the control group, while these alterations were reversed by the intervention of both low- and high-dosage LPLM141 (Figure 7(b)). We next examined the accumulation of fat in hepatic tissues by histological studies. The H&E (Figure 7(c)) and oil red O (Figure 7(d)) staining of the liver in the control group exhibited a normal histological architecture characterized by the negligible appearance of large vacuoles and minimal lipid accumulation (Figures 7(c) and 7(d), respectively, control group). Compared to the control group, high-fat diet treatment caused prominent diffuse macrovesicular and microvesicular steatosis (Figure 7(c), HFD group) and obvious oil red O staining in the liver (Figure 7(d), HFD group). However, both low- and high-dosage LPLM141 administration significantly improved the formation of hepatic steatosis (Figure 7(c), HFD + low- and high-dosage groups) and reduced hepatic lipid droplet accumulation (Figure 7(d), HFD + low- and high-dosage groups) induced by a high-fat diet feeding.

## 4. Discussion

Our current findings clearly showed that administration of both low- and high-dosage LPLM141 reduced body weight gain, liver weight, and adipose tissue weight without affecting the total amount of diet intake in high-fat diet treated rats (Table 3), indicating that this lowering effect was not explained by decreased food intake or loss of appetite but was associated with reduced food efficiency or increased energy expenditure evidenced by our current findings demonstrating that LPLM141 intervention facilitated energy expenditure and thermogenesis (Figure 4).

In this research, exposure of both low- and high-dosage LPLM141 decreased lipid accumulation in adipose tissues (Figure 1(c)) and the size of epididymal white adipocyte (Figures 1(a) and 1(b)) and suppressed serum total cholesterol, triglyceride, and LDL-C levels induced by high-fat diet treatment, whereas the downregulation of serum HDL-C concentration in the HFD group was reversed by both low- and high-dosage LPLM141 intervention (Figure 2). These results suggested that LPLM141 administration attenuated lipid accumulation in adipose tissue and relieved the disorder of lipid metabolism in high-fat diet-fed rats.

A systemic low-grade inflammation characterized by elevation of proinflammatory cytokines is usually observed in obesity. The levels of circulating LPS, adiponectin, and MCP-1 play a critical role in the development of inflammation and obesity [32–34]. In our present study, significantly increased serum levels of LPS and MCP-1 and a decreased serum level of adiponectin were noticed in the rats fed with a HFD. Administration of both low- and high-dosage LPLM141 obviously reversed these alterations (Figures 3(a)–3(c), respectively). Furthermore, the results from immunohistochemical staining in eWAT demonstrated that the increased macrophage infiltration in HFD-fed rats was dramatically decreased by both low- and high-dosage LPLM141 treatments (Figure 3(d)). It is now admitted that the expression of proinflammatory cytokines, namely, TNF-*α*, IL-6, and IL-1*β*, by infiltrated macrophages in adipose tissues could enhance and perpetuate the inflammation of adipose tissues often seen in obese humans and animals [35–37]. As demonstrated in the Figures 3(e) and 3(f) that correlated well with the macrophage infiltration in eWAT shown in the present results (Figure 3(d)), the mRNA expressions of TNF-*α*, IL-6, and IL-1*β* in epididymal and subcutaneous white adipose tissues in HFD-fed rats were significantly increased, whereas both low- and high-dosage LPLM141 intervention markedly suppressed this enhancing effect. In addition to proinflammatory cytokines, adipose tissue macrophages also produce a certain amount of PPAR-*γ*, a member of the nuclear hormone receptor family of transcription factors, to negatively regulate a large set of inflammatory pathway genes [26, 38]. The previous study demonstrated that addition of TNF-*α* downregulated PPAR-*γ* expression and ultimately contributed to inflammation in adipose tissues [39]. These results suggested that PPAR-*γ* is strongly correlated with the anti-inflammatory response in adipose tissue. We found that both low- and high-dosage LPLM141 intervention significantly increased the PPAR-*γ* mRNA expression in adipose tissue which was decreased in high-fat diet-fed rats (Figure 3(e)). Taken together, our results revealed that LPLM141 exerted its anti-inflammatory effects by decreasing serum LPS, MCP-1, proinflammatory cytokines, and infiltrated macrophages in eWAT and simultaneously increasing serum adiponectin and PPAR-*γ* expression in adipose tissues.

Obesity and its associated metabolic complications are always accompanied with insulin resistance. Our findings demonstrated that administration of both low- and high-dosage LPLM141 significantly prevented the development of hyperglycemia and ameliorated insulin resistance in HFD-induced obese rats (Figures 5(a)–5(e)). Leptin is an important hormone secreted by adipose tissue which regulates appetite and energy balance [40]. A large body of results from humans and animal studies indicated that elevated leptin levels were observed in obese hosts, especially in diet-induced obesity [40, 41]. Paradoxically, in our present finding shown in Figure 5(f), the increased serum leptin concentration in HFD-fed rats was not accompanied with the expected appetite reducing and body weight lowering effects (see Table 3). However, administration of both low- and high-dosage LPLM141 significantly downregulated the serum leptin levels upregulated by high-fat diet feeding (Figure 5(f)). This phenomenon can be explained by the occurrence of leptin resistance, which is defined by the reduced ability of leptin to suppress appetite and weight gains [42, 43]. More importantly, accumulating evidences demonstrated that the obese animals and humans who displayed leptin resistance may directly contribute to the suppression of lipid oxidation in insulin-sensitive organs and ultimately lead to accumulation of lipids and insulin resistance [40, 41, 44, 45]. Insulin resistance induced by obesity is characterized by a dysfunction of insulin to activate the IRS/phosphoinositide 3-kinase/AKT pathway, leading to suppression of the insulin-induced glucose uptake in the insulin-sensitive organs, such as the liver [46, 47]. In our present studies (Figure 6), high-fat diet treatment significantly downregulated IRS-1 and p-Akt^Ser473^ protein expressions in rat hepatic tissues, whereas administration of both low- and high-dosage LPLM141 effectively reversed the high-fat diet-induced decline of IRS-1 and p-Akt^Ser473^ (Figures 6(b) and 6(c), respectively). These results implied that LPLM141 intervention significantly mitigated insulin resistance by enhancing the IRS-1/p-AktSer473 insulin signaling pathway and lowering the serum leptin level in high-fat diet-fed rats and subsequently improving glucose intolerance and normalizing the HOMA-IR index.

Nonalcoholic fatty liver disease (NAFLD), a chronic liver disease characterized by excess fat deposition in the liver, is strongly associated with obesity and the metabolic syndrome [48]. Srebp-1c is an important transcription factor reported to be involved in the transcriptional activation of genes encoding rate-limiting enzymes in lipogenesis and is also associated with increased *de novo* lipogenesis in NAFLD [49]. Upon insulin stimulation, SREBP-1c regulates hepatic fatty acid and TG biosynthesis by upregulating the expression of key genes, such as FAS [50]. In addition, the previous study indicated that inhibition of Elovl6, a key lipogenic enzyme elongating long-chain saturated and unsaturated fatty acids, can ameliorate insulin resistance in fatty livers and suppress fatty acid synthesis [51]. Moreover, Elovl6 is regulated directly and primarily by SREBP-1c [52]. Our present results revealed that administration of both low- and high-dosage LPLM141 significantly counteracted the increase in liver lipogenic genes, namely, Elvol6, FAS, and Srebp-1c expressions in rats fed with HFD (Figure 7(a)). Administration with low- and high-dosage LPLM141 obviously attenuated steatosis and oil red O staining which were enhanced in liver tissue of HFD-fed rats (Figures 7(c) and 7(d)) and further corroborated their inhibitory effect on lipid accumulation. Several lines of evidence indicate that the animals fed with high-fat diet induce obesity accompanied by liver damage, similar to the phenotype observed in humans suffering from NAFLD [53]. In line with the previous studies, our present findings exhibited that a significant increase in serum AST and ALT was noticed in HFD-fed rats, whereas administration of both low- and high-dosage LPLM141 markedly alleviated these two liver damage markers induced by HFD treatment (Figure 7(b)). These results suggested that LPLM141 intervention exerted hepatoprotective activity via suppression of lipogenesis and fat accumulation.

Although our preliminary results supported LPLM141 effectively alleviating inflammation and insulin resistance in HDF-induced obese rats, some limitations are still needed to be mentioned here. First, it has been well documented that probiotics supplementation exerted its antiobesity effect by restoring the gut microbiota and mitigating metabolic endotoxemia by decreasing intestinal permeability [54–56]. Therefore, the impact on the composition of gut microbial populations and intestinal barrier integrity in HFD-induced obese rats after LPLM141 intervention will be explored. Second, for future long-term studies, the actual energy expenditure should be measured to consolidate our present findings indicating increased expression of genes encoding beige adipocyte activation and thermogenesis in eWAT and iBAT, respectively, in HFD-induced obese rats after LPLM141 treatment. Upon finishing these tasks, we can elucidate the mechanism of the antiobesity effects of LPLM141 more comprehensively.

## 5. Conclusion

In summary, we preliminarily proved that LPLM141 possessed an excellent antiobesity effect in a HFD-induced obese rat model. The potential mechanisms of this effect might be related to significantly attenuated body weight gain, reduced fat accumulation in adipose tissues, enhanced eWAT browning and iBAT activation, protected rats from glucose intolerance, and insulin resistance induced by HFD. These aforementioned effects were at least in part due to the anti-inflammatory actions of LPLM141 intervention in adipose tissues. Moreover, upregulation of serum adiponectin and downregulation of serum leptin concentrations after LPLM141 administration in HFD-fed rats might make contributions to alleviation of systemic inflammation and improvement of insulin resistance, respectively. We also further clarified that the underlying mechanism on improving insulin resistance after LPLM141 intervention was an attribute to the recoveries of IPS-1 and p-Akt expressions which were decreased in HFD-fed rats. Finally, our findings indicated that administration of LPLM141 remarkably ameliorated hepatic steatosis and exerted hepatoprotective effect in rats fed with a high-fat diet. Our current studies could offer LPLM141 as a promising probiotic for the prevention or therapy of obesity and its associated metabolic disorders.

## Figures and Tables

**Table 1:** Components of the standard diet and the high-fat diet.

Components (g/kg)	Standard diet	High-fat diet
Casein	199.8	231.7
_L_-Cystine	3.5	3.5
_D,L_-Methionine	N.D.	3.5
Corn starch	397.5	136.9
Maltodextrin	131.7	152.4
Sucrose	1002	162.6
Cellulose	48.2	48.2
Cholesterol	N.D.	1.9
Mineral mix (AIN-93)	35.8	40.6
Calcium phosphate dibasic	N.D.	4.6
Vitamin mix (AIN-93)	10.2	16.2
Choline bitartrate	2.5	5.1
tert-butylhydroquinone	0.02	0.04
Soybean oil	70.3	40.7
Lard	N.D.	155.5

N.D.: not detectable.

**Table 2:** Primer sequences used for the quantitative real-time PCR.

Genes	Forward	Reverse
Elvol6	GCCATGTTCATCACGTTGTC	CCGATGTAGGCCTCAAAGAA
FAS	GCTGCAAGCACAGCCTCTCT	GGCATCATTGGGCACTCCTT
Srebp-1C	AGTTTCTGGTTGCTGTGCTGTAAG	CGCTACCGTTCCTCTATCAATGAC
TNF-*α*	CAGCCGATTTGCCATTTCA	AGGGCTCTTGATGGCAGAGA
IL-6	TCCTACCCCAACTTCCAATGCTC	TTGGATGGTCTTGGTCCTTAGCC
IL-1*β*	GGGTTCCATGGTGAAGTCAAC	CACCTCTCAAGCAGAGCACAG
PPAR-*γ*	TGTGGACCTCTCTGTGATGG	CATTGGGTCAGCTCTTGTGA
Ucp-1	ACTGCCACACCTCCAGTCATT	CTTTGCCTCACTCAGGATTGG
Prdm16	CAGCACGGTGAAGCCATTC	GCGTGCATCCGCTTGTG
PGC1*α*	AGGACACGAGGAAAGGAAGAC	GGTAGCACTGGCTTGAATCTG
Cidea	TGCTCTTCTGTATCGCCCAGT	GCCGTGTTAAGGAATCTGCTG
Tmem26	ACCCTGTCATCCCACAGAG	TGTTTGGTGGAGTCCTAAGGTC
CD137	AGAAGCCTTGCTCCTCTACC	AACCCTGCTCCGTTAGTTCC
*β*-actin	TCTGTGTGGATTGGTGGCTCT	GACTCATCGTACTCCTGCTTGCT

**Table 3:** Effects of *Lacticaseibacillus paracasei* LM-141 on body weight, food intake, and tissue weights in HFD-induced rats.

Parameters/Groups	Control	HFD	HFD + low-dosage	HFD + high-dosage
Initial body weight (g)	312.00 ± 3.09	318.67 ± 2.07	316.67 ± 1.86	318.63 ± 1.68
Final body weight (g)	480.76 ± 9.83	546.60 ± 6.81^*∗*^	520.00 ± 4.87^**#**^	499.83 ± 4.19^**#**^
Body weight gain ratio	0.54 ± 0.02	0.72 ± 0.01^*∗*^	0.64 ± 0.01^**#**^	0.57 ± 0.01^**#**^
Food intake (g/day/rat)	23.92 ± 0.75	22.97 ± 0.17	22.69 ± 0.31	23.17 ± 0.35

*Adipose tissue weight* (*% of body weight*)				
sWAT (subcutaneous white adipose tissue)	1.91 ± 0.08	3.97 ± 0.14^*∗∗*^	2.53 ± 0.07^**#**^	2.26 ± 0.15^**#**^
eWAT (epididymal white adipose tissue)	2.54 ± 0.11	5.36 ± 0.09^*∗∗*^	3.81 ± 0.10^**#**^	3.37 ± 0.13^**#**^
iBAT (interscapular brown adipose tissue)	0.26 ± 0.03	0.23 ± 0.06	0.23 ± 0.03	0.25 ± 0.07
Liver weight (% of body weight)	2.82 ± 0.08	3.53 ± 0.01^*∗*^	2.68 ± 0.03^**#**^	2.47 ± 0.02^**#**^
Kidney weight (% of body weight)	0.80 ± 0.01	0.79 ± 0.01	0.80 ± 0.01	0.81 ± 0.01
Spleen weight (% of body weight)	0.17 ± 0.01	0.18 ± 0.03	0.17 ± 0.01	0.17 ± 0.04

^*∗*^ *p* < 0.05 and ^*∗∗*^*p* < 0.01 compared with the control group; #*p* < 0.05 compared with the HFD group.

## Data Availability

The datasets used or analyzed during the current study are available from the corresponding author upon reasonable request.
